# Prolonged Primary Cytomegalovirus Infection in an Immunocompetent Adult Successfully Treated With Valganciclovir

**DOI:** 10.7759/cureus.116320

**Published:** 2026-09-15

**Authors:** Sara K Iguchi, Shogo Shirota, Tomoki Kobayashi, Miwa Misawa

**Affiliations:** 1 General Medicine, Osaka Medical and Pharmaceutical University Hospital, Takatsuki, JPN

**Keywords:** cytomegalovirus (cmv) infection, fuo (fever of unknown origin), immunocompetent adult, infective mononucleosis, prolonged fever, valganciclovir

## Abstract

Cytomegalovirus (CMV) infection in immunocompetent adults is generally asymptomatic or causes a mild, self-limited illness, but it may occasionally present as CMV mononucleosis with prolonged fever. We report a case of primary CMV infection in an immunocompetent adult presenting with prolonged fever and subsequent clinical improvement following antiviral therapy. A 26-year-old woman presented with a three-week history of intermittent fever reaching 39°C. Laboratory findings revealed atypical lymphocytes and elevated liver enzymes. Serological findings were consistent with primary CMV infection. Despite supportive care, the fever and liver dysfunction persisted, and CMV antigenemia was subsequently found to be positive. Valganciclovir was initiated, and the patient became afebrile within five days, followed by improvement in the laboratory abnormalities. This case highlights that primary CMV infection in immunocompetent adults can cause prolonged fever lasting more than five weeks. In selected patients with persistent symptoms and evidence of ongoing viral activity, antiviral therapy may warrant consideration.

## Introduction

Cytomegalovirus (CMV) is a ubiquitous herpesvirus. CMV infection encompasses a wide clinical spectrum, including asymptomatic infection, mononucleosis-like syndrome, and organ-specific diseases such as colitis, hepatitis, pneumonitis, and retinitis [1]. CMV infection is generally considered a major clinical concern in immunocompromised hosts, in whom it is associated with serious end-organ disease [2].

In immunocompetent adults, primary CMV infection is usually asymptomatic or causes a mild, self-limited mononucleosis-like illness [3]. When symptomatic, CMV mononucleosis commonly presents with prolonged fever, malaise, lymphocytosis, and liver dysfunction [4,5]. The mean duration of fever has been reported to be 18 days; however, in some cases, fever persists for more than one month [6,7]. CMV mononucleosis is an important differential diagnosis in patients with fever of unknown origin (FUO), and an increasing incidence of CMV mononucleosis requiring hospitalization has been reported in recent years [8,9]. With serological and virological assays, including CMV antigenemia testing and PCR, now readily available, active CMV infection can be identified more promptly [1,8].

CMV infection in immunocompetent individuals is usually self-limited and managed without specific antiviral therapy [1,3]. However, some patients may experience prolonged symptoms or develop severe organ involvement, including central nervous system infection and pneumonia [10]. The role of antiviral therapy in such patients remains uncertain because established treatment guidelines for severe or prolonged CMV infection in immunocompetent individuals are lacking [3,5]. Moreover, ganciclovir and valganciclovir can cause clinically significant adverse effects, particularly hematologic toxicity and renal impairment [5,11]. Therefore, determining which patients should receive antiviral therapy and when treatment should be initiated remains challenging. Nevertheless, several reports have described favorable responses to ganciclovir or valganciclovir in selected patients [3,5,11-14].

Here, we report a case of primary CMV infection in an immunocompetent adult presenting with fever persisting for five weeks who experienced rapid clinical improvement after treatment with valganciclovir.

## Case presentation

A 26-year-old woman presented to our hospital with a three-week history of intermittent fever reaching 39°C. She also developed sore throat, nasal discharge, and cough 10 days prior to presentation. She was treated with multiple antibiotics and analgesics at several primary care clinics before presentation. Although the upper respiratory symptoms had improved, the fever persisted, and she was referred to our hospital and subsequently admitted. Her past medical history included a rectal polyp and atopic dermatitis treated with topical deprodone propionate. She had no known immunodeficiency, malignancy, history of organ transplantation, or use of immunosuppressive medications. She lived with her husband and her six-month-old daughter.

On admission, her vital signs were as follows: body temperature 38.1°C, blood pressure 112/66 mmHg, heart rate 84 bpm with a regular rhythm, respiratory rate 16 breaths/min, and oxygen saturation 99% on room air. On physical examination, there was no scleral icterus or conjunctival pallor. Palpable lymph nodes measuring 5-10 mm in diameter were noted in the bilateral posterior cervical and right axillary regions; they were soft, mobile, and non-tender. The abdomen was flat and soft without tenderness, and no hepatosplenomegaly was detected. Laboratory investigations revealed atypical lymphocytes, elevated liver enzymes (aspartate aminotransferase (AST) 58 U/L, alanine aminotransferase (ALT) 82 U/L), and an increased soluble interleukin-2 receptor level (728 U/mL; reference value: 121-613 U/mL). Serological testing showed a CMV IgM level of 0.95 AU/mL (reference value <0.85 AU/mL) and a CMV IgG level of 10.8 AU/mL (reference value <6.0 AU/mL). Serological testing for Epstein-Barr virus (EBV) showed negative results for viral capsid antigen (VCA) IgM, VCA IgG, and Epstein-Barr nuclear antigen. Blood and urine cultures were negative. Multiplex respiratory PCR testing showed no evidence of other common respiratory viral infections, and additional serological testing for toxoplasmosis, parvovirus B19, mumps, rubella, HIV, hepatitis B, and hepatitis C was negative. Non-contrast CT from the neck to the abdomen revealed multiple enlarged lymph nodes in the cervical and axillary regions, with the right axillary lymph node being the most prominent (Figure 1), but no other significant abnormalities or splenomegaly were observed. These findings suggested infectious mononucleosis caused by primary CMV infection. Lymph node biopsy of the right axillary node was also considered to exclude malignant lymphoma; however, the patient declined the procedure.

**Figure 1:**
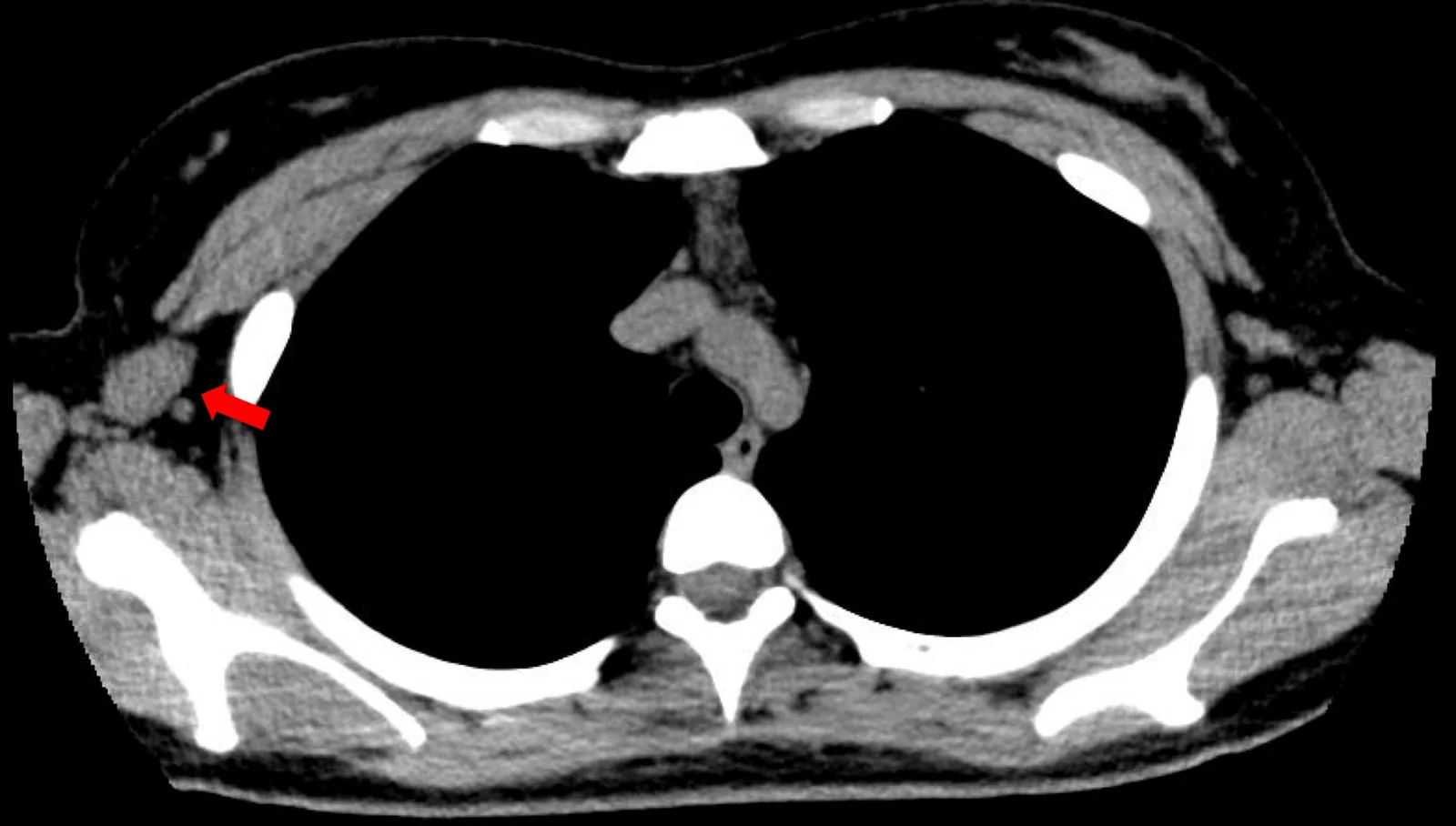
Computed tomography of the chest. Non-contrast computed tomography of the chest showing the most prominent enlarged right axillary lymph node (arrow).

After admission, antibiotics and other nonessential medications were discontinued, and the patient received supportive care with acetaminophen and loxoprofen for two weeks; however, she remained febrile, with temperatures of approximately 38°C, and liver enzyme levels increased (AST 144 U/L, ALT 223 U/L). Serological testing showed that the CMV IgM level had increased to 25.2 AU/mL and the CMV IgG level to 31.5 AU/mL. CMV antigenemia measured by the C7-HRP assay was positive, with nine positive cells per 50,000 leukocytes. The marked increase in CMV IgM and IgG levels, together with positive CMV antigenemia and the compatible clinical presentation, further supported the diagnosis of active CMV infection, most consistent with primary CMV infection. Given the persistence of fever for approximately five weeks, progressive worsening of liver dysfunction, and positive CMV antigenemia, ongoing CMV activity was suspected. Therefore, treatment with valganciclovir at a dose of 900 mg every 12 hours was started. The patient became afebrile within five days. Follow-up CMV antigenemia testing was negative, and valganciclovir was discontinued after six days of treatment. Ten days after initiation of valganciclovir, follow-up laboratory tests showed improvement in liver enzyme levels (AST 45 U/L, ALT 101 U/L). No adverse events attributable to valganciclovir were observed during treatment or at follow-up. The laboratory findings at admission and during the subsequent clinical course are summarized in Table 1. The clinical course is summarized in Figure 2.

**Table 1:** Laboratory findings during the clinical course. AST: Aspartate aminotransferase; ALT: Alanine aminotransferase; GGT: Gamma-glutamyl transferase; ALP: Alkaline phosphatase; LDH: Lactate dehydrogenase; sIL-2R: Soluble interleukin-2 receptor; CMV: Cytomegalovirus; EBV: Epstein-Barr virus; VCA: Viral capsid antigen; EBNA: Epstein-Barr nuclear antigen.

Parameter	On admission	Two weeks after admission	Follow-up	Reference range
WBC	4,260 /μL	-	-	3,300-8,600 /μL
Band neutrophils	9.50%	-	-	0-5.0%
Segmented neutrophils	49.00%	-	-	35.0-68.0%
Lymphocytes	28.00%	-	-	22.2-50.9%
Atypical lymphocytes	5.50%	-	-	0%
Hemoglobin	13.6 g/dL	-	-	13.7-16.8 g/dL
Platelets	203 × 10³/μL	-	-	158-348 × 10³/μL
Total bilirubin	0.2 mg/dL	-	-	0.4-1.5 mg/dL
AST	58 U/L	144 U/L	45 U/L	13-30 U/L
ALT	82 U/L	223 U/L	101 U/L	10-42 U/L
GGT	67 U/L	-	-	13-64 U/L
ALP	108 U/L	-	-	38-113 U/L
LDH	269 U/L	-	-	124-222 U/L
CRP	0.2 mg/dL	-	-	0.00-0.14 mg/dL
Soluble IL-2 receptor	728 U/mL	-	-	121-613 U/mL
CMV IgM	0.95 AU/mL	25.2 AU/mL	-	<0.85 AU/mL
CMV IgG	10.8 AU/mL	31.5 AU/mL	-	<6.0 AU/mL
CMV antigenemia (C7-HRP)	-	9 positive cells/50,000 leukocytes	Negative	Negative
EBV VCA IgM	<10	-	-	<10
EBV VCA IgG	<10	-	-	<10
EBV nuclear antigen (EBNA)	<10	-	-	<10

**Figure 2:**
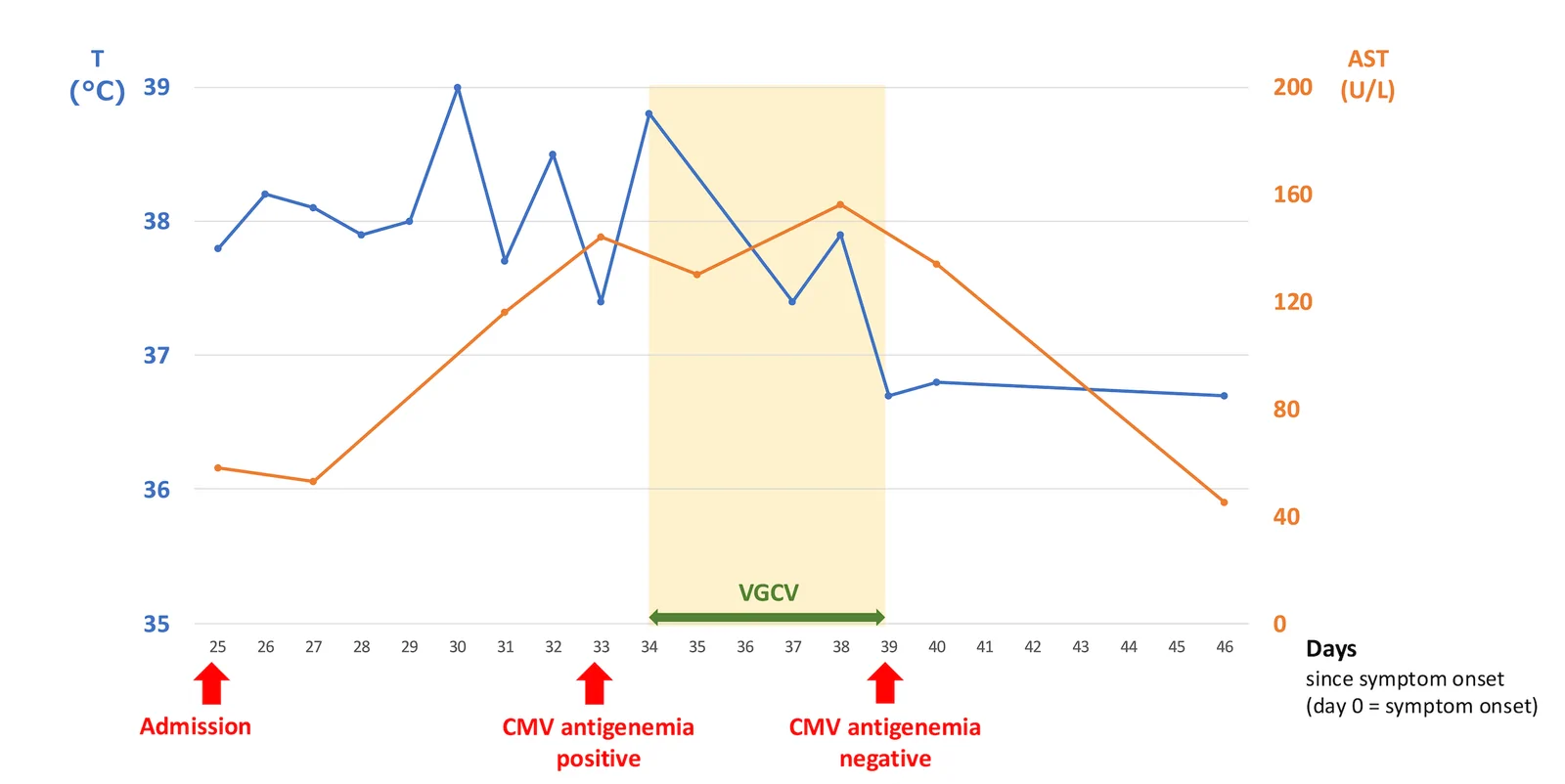
Clinical course of the patient. The figure shows the clinical course from hospital admission, including changes in body temperature and liver enzyme levels, CMV antigenemia results, and the duration of valganciclovir therapy. Day 0 was defined as the day of symptom onset. T: Temperature; CMV: Cytomegalovirus; VGCV: Valganciclovir; AST: Aspartate aminotransferase.

## Discussion

This case illustrates two important clinical features: primary CMV infection in immunocompetent adults can cause prolonged fever lasting more than five weeks, and, in this patient, defervescence occurred shortly after initiation of valganciclovir.

Although symptomatic CMV mononucleosis in immunocompetent adults commonly causes prolonged fever, most patients improve within four weeks [4,5,7]. Fever persisting for five weeks or longer appears to be uncommon, and previously reported cases with such a prolonged course are summarized in Table 2. In some cases, the clinical course fulfilled the classical criteria for FUO, defined as a temperature ≥38.3°C on several occasions for more than three weeks, with no diagnosis established after at least one week of inpatient investigation [4,9,15]. In cases presenting with FUO, findings such as lymphocytosis, the presence of atypical lymphocytes, liver enzyme disturbances, and a history of contact with young children may raise suspicion for primary CMV infection [1,4,8].

**Table 2:** Reported cases of CMV infection with a mononucleosis-like presentation and prolonged fever in immunocompetent adults. CMV: Cytomegalovirus; M: Male.

Reference	Age/Sex	Symptoms and notable features	Duration of fever
[5]	35/M	Fever, elevated liver enzymes	5 weeks
[7]	66/M	Fever, weight loss, hepatosplenomegaly	9 weeks
[7]	32/M	Fever, weight loss, splenomegaly	10 weeks
[7]	35/M	Fever, weight loss, hepatosplenomegaly	8 weeks
[7]	32/M	Fever, weight loss, splenomegaly	11 weeks

Antiviral treatment is well established for CMV disease in immunocompromised patients [1]. However, its role in CMV mononucleosis in immunocompetent adults remains uncertain. In previously reported cases of CMV mononucleosis in immunocompetent adults with persistent or relapsing symptoms, initiation of ganciclovir or valganciclovir was followed by rapid clinical improvement, with defervescence occurring within several days (Table 3) [5,13]. Similarly, in the present case, defervescence occurred within five days after initiation of valganciclovir, suggesting a possible clinical benefit of antiviral therapy in carefully selected immunocompetent patients with persistent symptomatic CMV infection.

**Table 3:** Reported cases of symptomatic CMV infection with a mononucleosis-like presentation treated with antiviral therapy in immunocompetent adults. CMV: Cytomegalovirus; M: Male; F: Female; w: Week(s); IgM: Immunoglobulin M; VGCV: Valganciclovir; GCV: Ganciclovir.

Reference	Age/sex	Symptoms and notable features	Diagnosis	Treatment	Outcome
[5]	52/M	2 w of fever, neck stiffness, splenomegaly; atypical lymphocytosis, elevated liver enzymes	CMV IgM and CMV DNA PCR (blood)	VGCV	Defervescence and improvement in headache within 48 hours
[5]	35/M	5 w of fever; elevated liver enzymes	CMV IgM and CMV DNA PCR (blood)	VGCV	Defervescence within 48 hours
[5]	72/M	2 w of low-grade fever; leukocytosis with lymphocyte predominance, elevated liver enzymes	CMV DNA PCR (blood)	VGCV (10 days)	Defervescence and clinical improvement within 5 days
[13]	31/F	2 w of fever, myalgia, abdominal pain, hepatosplenomegaly; atypical lymphocytosis, elevated liver enzymes	CMV IgM and CMV antigenemia	GCV (IV for 5 days → oral for 3 days)	Defervescence and clinical improvement within 2 days
Present case	26/F	5 w of fever, lymphadenopathy; elevated liver enzymes	CMV IgM and CMV antigenemia	VGCV (6 days)	Defervescence within 5 days; improvement in liver enzyme levels after 10 days

Regarding the indications for antiviral therapy in immunocompetent adults with CMV infection, clear criteria have not been established, and treatment decisions should be individualized based on clinical severity and disease course. Previous reports have used persistent or worsening symptoms together with detectable CMV in the blood as factors supporting the initiation of antiviral therapy (Table 3) [5,13]. We also considered CMV antigenemia as evidence of ongoing viral activity, drawing on the preemptive approach used in hematopoietic stem cell transplant recipients [16]. In the present case, antiviral therapy was initiated because fever persisted for five weeks, liver dysfunction progressively worsened, and CMV antigenemia was positive. Based on previous reports and the present case, antiviral treatment may be considered in cases with persistent or worsening symptoms, particularly when accompanied by evidence of ongoing viral activity or worsening hepatic dysfunction.

The duration of ganciclovir or valganciclovir therapy has varied among previously reported cases [3,5,10-14], and the optimal duration remains undefined. In the present case, the treatment duration was determined with reference to management strategies used in hematopoietic stem cell transplant recipients, in whom discontinuation of antiviral therapy is generally guided by both resolution of clinical symptoms and clearance of virological markers [16]. Accordingly, valganciclovir was discontinued on day 6 of therapy after defervescence and confirmation of CMV antigenemia clearance.

This case report has several limitations. Quantitative CMV DNA PCR was not performed, and virological assessment relied on CMV antigenemia. In addition, long-term clinical, laboratory, and virological follow-up after discontinuation of valganciclovir was not available. Finally, given the generally self-limiting nature of CMV infection in immunocompetent individuals, spontaneous resolution cannot be excluded, and the temporal association between valganciclovir initiation and clinical improvement does not establish causality.

## Conclusions

We described a case of primary CMV infection in an immunocompetent adult in whom fever persisted for five weeks and resolved shortly after the initiation of valganciclovir. This case highlights that CMV infection in immunocompetent adults can present with prolonged fever and suggests a potential benefit of antiviral treatment. Further studies are needed to clarify the natural history of prolonged CMV mononucleosis and the indications for and optimal duration of antiviral therapy.
